# Potent Neutralizing Humanized Antibody With Topical Therapeutic Potential Against HPV18-Related Cervical Cancer

**DOI:** 10.3389/fimmu.2021.678318

**Published:** 2021-06-24

**Authors:** Bilian Huang, Linjing Zhu, Hongxia Wei, Haixia Shi, Doudou Zhang, Huanyun Yuan, Linlin Luan, Nan Zheng, Shijie Xu, Waqas Nawaz, Ying Hong, Xilin Wu, Zhiwei Wu

**Affiliations:** ^1^ Center for Public Health Research, Medical School, Nanjing University, Nanjing, China; ^2^ Department of Antibody, Abrev Biotechnology Co., Ltd., Nanjing, China; ^3^ Department of Infection, Nanjing Hospital Affiliated to Nanjing University of Chinese Medicine, Nanjing, China; ^4^ Department of Antibody, Y-Clone Medical Science Co. Ltd., Suzhou, China; ^5^ Obstetrics and Gynecology Department, Nanjing Drum Tower Hospital, Affiliated Hospital of Nanjing University Medical School, Nanjing, China; ^6^ School of Life Sciences, Ningxia University, Yinchuan, China; ^7^ Jiangsu Key Laboratory of Molecular Medicine, Medical School, Nanjing University, Nanjing, China; ^8^ State Key Laboratory of Analytical Chemistry for Life Science, Nanjing University, Nanjing, China

**Keywords:** HPV18, cervical cancer, neutralizing antibodies, topical agents, humanized antibody

## Abstract

Cervical cancer caused by human papillomavirus (HPV) infections is the fourth most common cancer in women worldwide. Current prophylactic HPV vaccines have achieved promising success in preventing HPV infection. However, still 570,000 new cases were reported in 2018. The current primary treatment for the patient with cervical cancer is either surgery or chemoradiotherapy. Cervical cancer still lacks standard medical therapy. HPV18 induced cervical cancer has the worst prognosis and high mortality compared to other HPV infections. The development of HPV18 related with cervical malignancy requires the persistent infection of cervical–vaginal epithelium by HPV18 subtype, which can take years to transform the epithelium. This period of repeated infection provides a window for therapeutic intervention. Neutralizing antibodies formulated as topical agents that inhibit HPV18 infection should reduce the chance of cervical malignancy. We previously demonstrated that potent neutralizing anti-sera against HPV18 infection were induced by HPV18 viral like particle (VLP) generated in mammalian cells. We, therefore, isolated two potent neutralizing antibodies, 2A12 and 8H4, from over 3,810 hybridomas prepared from mice immunized with HPV18 VLP. 2A12 and 8H4 exhibited excellent potency, with 50% virus-inhibitory concentrations (IC_50_) of 0.4 and 0.9 ng/ml, respectively. Furthermore, 2A12 and 8H4 recognized distinct and non-overlapping quaternary epitopes and bound specifically with HPV18. Humanized 2A12 (Hu2A12) retained comparable neutralizing activity against HPV18 infection in various acidic pH settings and in hydrogel formulation with IC_50_ values of 0.04 to 0.77 ng/ml, indicating that Hu2A12 will be a promising candidate for clinical development as a topical vaginal biopharmaceutical agent against HPV18 infection.

**Graphical Abstract:**
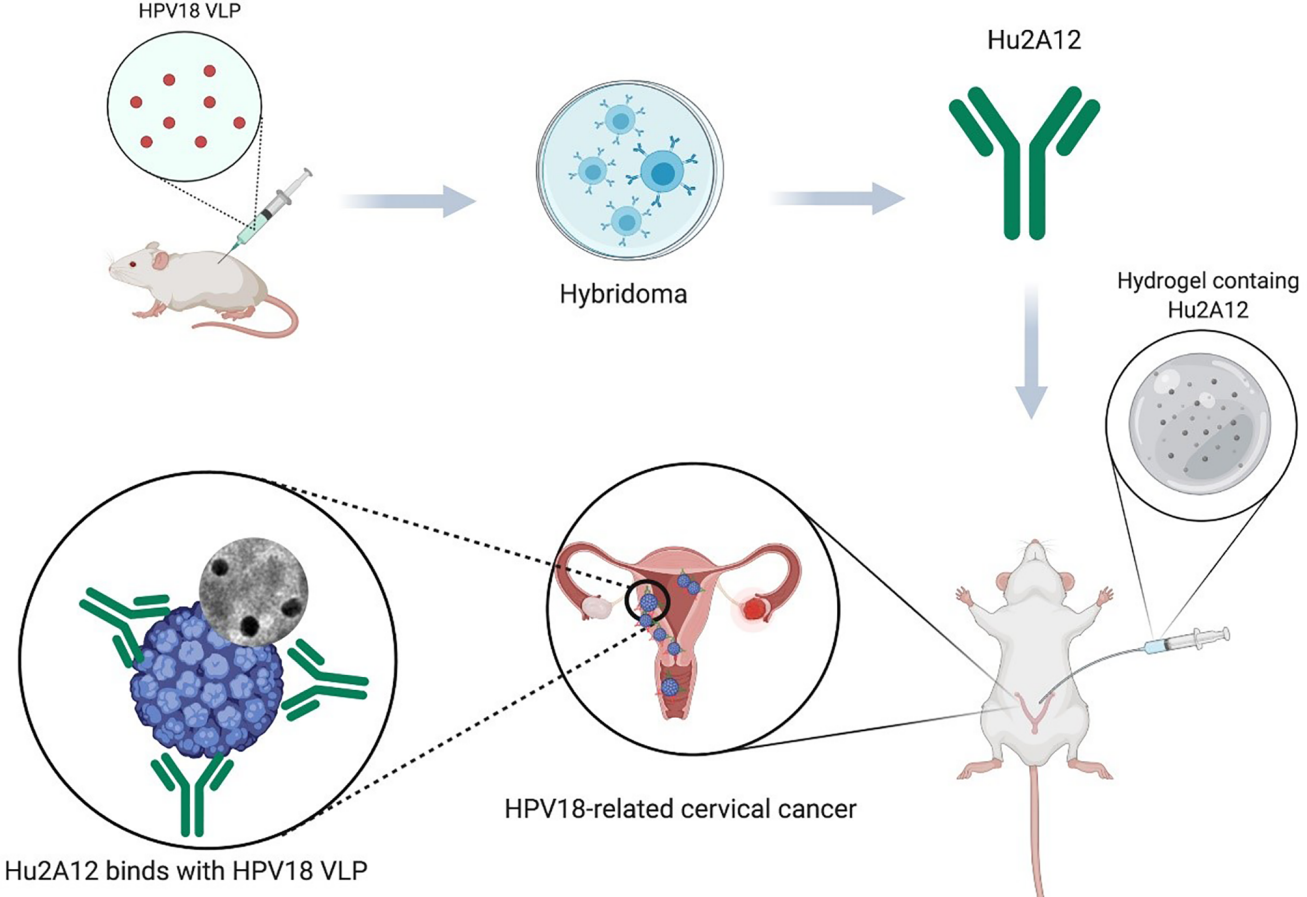


## Highlights

1. Two neutralizing antibodies against HPV18 with the highest potency were isolated from immunized mice.
2. The humanized antibody, Hu2A12, exhibits ultrahigh potency against HPV18 infection with IC_50_ value of 0.04 ng/ml.
3. Hu2A12 retains comparable neutralizing activity in various acidic pH settings and in hydrogel formulation.
4. Hu2A12 will be a promising candidate as a topical vaginal biopharmaceutical agent against HPV18 infection.

## Introduction

Cervical cancer is the fourth most common tumor diagnosed in women worldwide (1). Persistent infections caused by high-risk Human papillomaviruses (HPV), such as 16, 18, 31, 33, 35, 39, 45, 51, 52, 56, 58, 59 and 68 are considered to be the main cause for the development of cervical cancer precursors, known as cervical intraepithelial neoplasia (CIN 1, 2, and 3), and invasive cervical cancer (2, 3).

Current prophylactic HPV vaccines have achieved remarkable success in preventing HPV infection (4, 5). However, a large number of women still failed to receive prophylactic HPV vaccines due to the high cost, failed to respond to the vaccination and other factors. In 2018, 570,000 new cases and 311,000 related deaths were reported globally, indicating effective treatment was urgently needed (6). Surgical or chemoradiotherapeutic regimens are the current primary treatment options for patients with cervical cancer. Specific medical treatment for HPV infection remains elusive (7).

Persistent HPV infection in the basal layer of the cervical epithelium is the main risk factor in the development of the premalignant conditions of cervical intraepithelial neoplasia or adenocarcinoma in situ. Without treatment, the transition from dysplasia to invasive carcinoma may take years to decades to develop in most women.

Current research on topical therapies for the treatment of HPV or CIN have promising results, signified by the randomized trials of immune-modulating (imiquimod), anti-proliferative (5-fluorouracil), and anti-viral (cidofovir) therapies (8–10). However, none of them has profound clinical evidence to be recommended as a treatment for CIN 2–3 and surgery remains the standard of care (11). Cidofovir is an approved antiviral drug for the treatment of cytomegalovirus (CMV) retinitis in HIV patients. Clinical studies of cidofovir gel as a topical therapy had shown promising results for clearance of HPV or CIN. However, serious side effects were recently reported in the use of cidofovir in the treatment of HPV infection (12). All these indicate that topical anti-viral therapy is promising; however, antibody based topical therapy for HPV or CIN has not been reported yet.

The use of neutralizing antibodies with high potency and low toxicity have been widely applied to treat viral infections caused by a respiratory syncytial virus, cytomegalovirus, human immunodeficiency virus, Ebola virus and influenza virus (13, 14). The discovery and development of virus-neutralizing monoclonal antibodies can be a promising approach to eliminate HPV persistent infection and prevent the subsequent transition of invasive carcinoma.

After HPV16, HPV18 infection is the second most carcinogenic HPV genotype in a large percentage (approximately 10%), and highly enriched in adeno/adenosquamous and adenocarcinoma *in situ* compared to lower grades of diagnosis (2, 15, 16). HPV18 causes cervical cancer with the worst prognosis as compared with other types of HPV (17, 18). Two neutralizing antibodies against HPV18 were previously developed for diagnostic kit for HPV-18 (19). To our knowledge, no neutralizing antibodies with high potency have been developed for HPV18 treatment. We previously demonstrated that high potent neutralizing anti-sera against HPV18 infection could be induced by our HPV18 viral like particle (VLP) generated in the mammalian cell. In this study, two potent neutralizing antibodies, 2A12 and 8H4, were isolated from more than 3,810 hybridomas prepared from mice immunized with HPV18 VLP (20). 2A12 and 8H4 exhibited high potency, with IC_50_ of 0.4 and 0.9 ng/ml, respectively. Furthermore, 2A12 and 8H4 recognized distinct and non-overlapping quaternary epitopes and exhibited specific binding with HPV18. Humanized 2A12 (Hu2A12) retains comparable neutralizing activity against HPV18 infection in various acidic pH conditions and hydrogel with the IC_50_ of 0.04 to 0.7 ng/ml, indicating that Hu2A12 will be a promising candidate for clinical development as topical vaginal biopharmaceutical agents in hydrogel against HPV18 infection.

## Methods and Materials

### Production of HPV18 VLP and HPV18 Pseudovirus

HPV18 VLP and pseudovirus were prepared using the plasmid of p18sheLL (Addgene, 37321) and the co-transfection of p18shell and pGMCMV-luc (Yeasen Biotech) as a reporter gene, respectively, as we previously described (20), with some modifications. In brief, the plasmids were mixed with PEI (Polysciences, MW25000, 23966-1) at a 1:3 ratio and then transfected into 293 TT cells. About 48 h post-transfection, cells were harvested and washed with DPBS twice. After centrifugation, the cell pellet was resuspended in 0.5% Triton X-100 and 25 mM ammonium sulfate. The cell lysate was then incubated at 37°C for 24 h. The matured lysate on ice was incubated for 15 min with the final concentration of 850 mM NaCl. After centrifugation, the supernatant was transferred for OptiPrep (D1156, Sigma-Aldrich, St. Louis, Missouri, USA) gradient purification at 40,000 rpm for 4.75 h. Different fractions were collected for verification.

### Immunization of Mice

Five 8-week-old female Balb/c mice (M1~M5) were immunized by three subcutaneous injections, at two weeks interval, of 25–50 μg HPV18 VLP emulsified with Freund’s adjuvant (Sigma, F5881 & F5506). Sera were collected one week after the third immunization and were used to further analysis. An intraperitoneal injection of HPV18 VLP without adjuvant was performed as the booster immunization before hybridoma.

### Enzyme-Linked Immunosorbent Assay

To measure the serum titer or antibody against HPV18 VLP, ELISA plates (Corning, 9018) were coated with 0.5 μg/ml purified HPV18 VLP at 4°C overnight, then blocked in 2% bovine serum albumin-PBST. Sera were diluted in 2% bovine serum albumin-PBST. Diluted sera or hybridoma culture supernatant was added and then incubated at 37°C for 1.5 h. After a wash with PBST, anti-mouse IgG HRP conjugated antibodies (Jackson ImmunoResearch115-035-003) were added at 37°C for 60 min. For visualization, 100 μl 3,3′,5,5′-Tetramethylbenzidine (TMB, Sigma) substrate was added and incubated at room temperature for 10 min, and stopped with 50 μl 1 M HCl per well. Optical densities were determined at 450 nm using Infinite 200 (Tecan).

### Preparation of Hybridomas and Identification of Candidate Clones

Mice were sacrificed 3 days after booster immunization. Splenocytes were isolated, fused with Sp2/0 cells (ATCC) in a 1:2 ratio using Electro Cell Manipulator (BTX Harvard Apparatus, ECM 2001) as described (21, 22). Fused cells were cultured in hypoxanthine aminopterin thymidine (HAT, Sigma, H0262) medium in multiple 96-well plates, and supernatants were tested after 7 days in culture. The supernatants of the individual hybridoma clones were screened for HPV18 VLP specific antibodies by ELISA. The selected positive hybridoma supernatants were further analyzed using neutralization assay. All the candidate clones were subcloned at least twice by limiting dilution.

### HPV Neutralization Assay

The pseudovirus-based neutralization assay was performed to evaluate the activity of antibodies inhibiting HPV infection, following the previously reported method (20) with slight modifications. Briefly, 10,000 293TT cells were seeded per well overnight in a 96-well cell culture microplate in growth media. Serial dilutions of antibodies or anti-sera were incubated with HPV pseudovirus at 37°C for 1 h. Then, the mixture was transferred to the 96-well plates and incubated with cells at 37°C for 48 h. After that, the supernatant was removed, and substrate (Promega Bright-Glo luciferase Assay System, E2650) was added to the samples to read Fluorescence intensity. The IC_50_ value was calculated based on the previously published standard algorithm (23).

### Western Blot

About 200 ng HPV VLPs were mixed with 5× SDS-PAGE non-reduce sample buffer (250 mM Tris–HCl, pH 6.8, 10% SDS, 0.05% bromophenol blue, 20% glycerol) and boiled for 5 min. For reduced HPV18 VLP, 5x SDS-PAGE reduce sample buffer (250 mM Tris-HCl, pH 6.8, 500 mM DTT, 10% SDS, 0.05% bromophenol blue, 20% glycerol) was added. The proteins were separated by electrophoresis on 10% SDS-PAGE gels, and the gels were then transferred onto a 0.45-μm PVDF membrane. (Bio-Rad, 10485196). The membrane was soaked in blocking buffer (2% BSA in PBST) at room temperature for 1 h, and then incubated with hybridoma supernatant or anti-sera at room temperature for 2 h, followed by three times of washing. A secondary antibody of anti-mouse IgG with an IRDye 800CW (Li-COR, 925-32210) was used and the immunoblots were visualized using an Odyssey Infrared Imaging System (LI-COR).

### Identification of Antibody Isotypes

Mouse antibody isotypes were determined using mouse monoclonal antibody subtype identification Kit (Cellway-Lab, C030215), following the manufacturer’s protocol. In brief, a sample of evaluated antibody was added in the ELISA plate. After incubation and washing, the secondary antibodies specific for IgG1, IgG2a, IgG2b, IgG3, IgA and IgM were added. The high optical density (450 nm) suggests the right antibody isotype or subtype.

### Flow Cytometric Analysis

293TT cells were transfected with the plasmid of p18sheLL to express VLP. Some 48 h later, cells were collected, fixed in formalin for 20 min, treated with 0.2% TritonX-100 for 10 min and then blocked in PBS with 1% FBS (PBSF) for 2 h. 2A12 and 8H4 were added and incubated for 1 h at room temperature. After washing twice with PBSF, binding antibodies were detected by an incubation at 4°C for 30 min with Alexa Fluor^®^ 488-AffiniPure Goat Anti-Mouse IgG (H + L) (Jackson Immunoresearch, 115-545-146). After washing, the cells were resuspended in 500 µl PBSF and analyzed using ACEA NovoCyte TM (Agilent Biosciences) Nontransfected 293TT cells were served as a negative control.

### Electron Microscopy and Immunogold Labeling

Immunoelectron microscopy (Immune-EM) of the HPV virion was performed as described previously (24). For staining, 10 μl HPV18 VLP was adsorbed on copper grids for 10 min. Afterward, the grids were blotted dry with filter paper and transferred facing down onto a drop of 2% BSA-PBS.10 min later, the grid floated on droplets including primary antibody for 1 h at room temperature, followed by the grids being rinsed on three drops of PBS in the same way. Then, an anti-mouse secondary antibody conjugated to 10 nm gold particles (Sigma G7652-.4ML) diluted 1:500 in 2% BSA-PBS was applied for 30 min. Following washing with PBS, the grid was negatively contrasted with 2% phosphotungstic acid (Macklin P829844-5g) by incubating at room temperature for 4 min. Finally, samples were dry and observed using transmission electron microscopy (JEM-2100).

### Sequencing and Analysis of Mouse Ig Genes

Some 1 ∗ 10^7^ hybridoma cells were collected and washed with PBS, then lysed with Trizol (Ambion, 15596018) to extract total RNA according to the manufacturer’s instructions. RNA was reverse transcribed to cDNA using PrimeScript™II 1st Strand cDNA Synthesis Kit (Takara, 6210A). The resulting cDNA was used as the template for amplifying heavy and light-chain variable gene using the Mouse Ig-Primer Set (Merck Millipore, 6983). The gene was then sequenced, adopting standard methods (25). The International ImMunoGeneTics Information System (IMGT) (http://imgt.cines.fr) was used to analyze the variable domain VH/VL. To confirm the sequences, the vectors of pCDNA3.4-VH-CH and pCDNA3.4-VL-CL containing the gene of VH and VL were constructed and co-transfected 293TT cells. The cell supernatant was then detected by ELISA as described above.

### Antibody Humanization

The mouse IgG humanization was achieved by complementarity determining regions (CDR) grafting (26, 27). The sequence of 2A12 VH/VL were blasted against human heavy and light variable chain by using searches of IMGT/Domain Gap Align comprehensive database of Ig. Combined with the sequence analysis of the Molecular Operating Environment (MOE) and its three-dimensional structure from molecular modeling, critical diversity residues in the framework of 2A12 were identified. Then CDR grafted with humanized heavy and light chain were constructed by partly replacing critical diversity residues with human original residues. The combined expression of diverse designed humanized heavy and light chains resulted in multiple different pairings of humanized antibodies molecules. Pairing with the highest affinity and neutralizing activity were selected.

### Quantification of Mouse IgG

The mouse IgG in serum or in hybridoma supernatant was quantified using double antibody sandwich ELISA. Briefly, the anti-mouse IgG (Sigma, M0659, Fab specific, F(ab′)_2_ fragment antibody produced in goat) was coated. After blocking, series gradient diluted sample and mouse IgG standards (0–100 ng/ml) were added as primary antibodies, while anti-mouse IgG (Fc specific)–peroxidase antibody (Sigma, A2554) was used as a secondary antibody. The linear regression curve of the mouse IgG standards was drawn, and the OD_450_ value was brought into the equation to calculate the mouse IgG concentration of each sample.

### Biolayer Interferometry

The affinity of HPV18 specific antibodies was determined using the Octet RED96 instrument (Sartorius). Amine reactive biosensor (AR2G, 18-5092) was used to immobilize the HPV18 VLP. AR2G biosensors were activated for 7 min with EDC (400 mM)/NHS (100 mM), and then immersed 15 min in HPV18 VLP which was diluted in pH 5.0 10 mM sodium acetate buffer. Transfer the sensor to ethanolamine for 7 min and then to 0.02% PBST for affinity assay. The kinetics assays were performed with a shaking speed of 1,000 rpm. Association with HPV18 VLP specific antibodies was measured for 4 min and dissociation in 0.02% PBST for 4 min. The affinity analysis was performed using a fast 1:1 binding model and the Data analysis software 8.0 (Sartorius).

The epitope-binding assay was performed with AR2G biosensor following the manufacturer’s protocol ‘in-tandem assay’. The immobilization of antigen was performed as above described, and then associated the first antibody (20 μg/ml) for 400 s following with the baseline step with 30s immersion in 0.02% PBST. Accordingly, the sensors were immersed for 400s with a second antibody at 20 μg/ml. Graph Pad was used to illustrate the time-response course of two antibodies binding to HPV18 VLP.

### Preparation of Antibody Hydrogel and *In Vitro* Release Kinetics

To generate a hydrogel formulation contained antibodies, Hu2A12 was diluted in sterile ddH_2_O at a final concentration of 1 mg/ml. Subsequently, 70 mg Hydroxyethyl cellulose (HEC, Macklin, H810926) was added to the 1 ml above antibody solution containing Hu2A12, which shook for 1 h at room temperature, accordingly, 7% (wt/vol) HEC hydrogel containing antibodies was prepared.

For drug release studies, 7% HEC containing Hu2A12 labeled with FITC (hu2A12-FITC) was prepared (28–30). Transwell (NEST, 725301) inserts perforated with ten 22-G needle were used as filters. About 300 μl hydrogel was applied onto the insert membrane, followed by a gentle addition of 0.5 ml PBS. Some 1.5 ml PBS was applied to the lower chamber. About 100 μl aliquots in the lower chamber were transferred to a black 96-well plate (Greiner, 655076) and made up by 100 μl fresh PBS at the indicated time. Hu2A12 labeled by FITC was quantified by fluorescence at 485 nm excitation wavelength and 525 nm emission wavelength using a fluorescence multi-well plate reader (Molecular Devices M3). *In vitro* cumulative percentage release was calculated using the formula as below:

$$\text{Relative amount of drug in released solution}=\text{O}{\text{D}}_{525}\;\text{of sample}\;\mathrm{withdrawn}*20$$

$$\text{Cumulative percentage release}\;(\%)=\frac{\text{relative}\;\text{amount}\;\text{of}\;\text{drug}\;\text{in}\;\text{released}\;\text{solution}\;\text{at}\;\text{time}\;\text{t}+\mathrm{cumulative}\;\text{drug}\;\text{withdrawn}\;\text{previous}\;\text{to}\;\text{t}}{\text{relative}\;\text{total}\;\text{amount}\;\text{of}\;\text{drug}}$$

### Neutralization Activity Under Acidic Conditions

To apply the antibodies under acidic conditions, we firstly detected the neutralization activity of Hu2A12 in the media of pH 4, 5, 6, and 7. The complete DMEM media was adjusted with acidic acid to pH 4, 5, 6, and 7. The neutralization assay was similar to the ‘HPV neutralization assay’ as described above with some changes as follow. Both antibodies and pseudovirus were diluted in corresponding acidic media. Four hours after adding the mixture of antibodies and pseudovirus in the seeded cells, the acidic media was replaced with conventional media to avoid the cells from acid toxicity. Some 48 h later, neutralizing activity was measured.

The Hu2A12 released from vaginal hydrogel was evaluated for neutralization activity. Sodium acetate buffer (pH5.0) was applied as an antibody released buffer to mimic the acidic environment of the vagina. About 1.5 fold volume of acidic buffer was added to the antibody hydrogel, making the gel disintegrated completely in 37°C, which was maintained for 72 h. At 24 and 72 h, the released antibodies were sampled for neutralization assay.

### *In Vivo* Retention of Antibody Hydrogel in a Mouse Model

Hu2A12 was labeled with far infrared dye YF^®^750 SE (US EVERBRIGHT INC, YS0056) (named Hu2A12-750). Nine female nude mice (18–22 g, Qing Long Shan Animal Breeding Grounds, Nanjing, China) were divided into three groups. Three mice in Group 1 were intra-vaginally treated with 50 μg Hu2A12-750 hydrogel, while three mice in Group 2 were injected with 50μg liquid Hu2A12-750 in the same way. The remaining three mice were used as negative control. Far infrared images were observed at 15 min, 4 h, 8 h, 24 h and 48 h with a small animal imaging system (NightOWL LB 983 NC100) at Ex: 740 nm/Em:780 nm. Images were captured by the CCD camera embedded in the imaging system and analyzed using Indigo imaging software Ver. A 01.19.01.

### Statistics

Graphs were generated by GraphPad Prism 5.01 software or OriginPro 8.5 software (Origin-Lab). One- or 2-way ANOVA was performed for group comparisons. P <0.05 was considered statistically significant with data shown as mean ± SEM or mean ± SD or median + range.

## Results

### Generation of Anti-HPV18VLP mAbs

**Figure 1:**
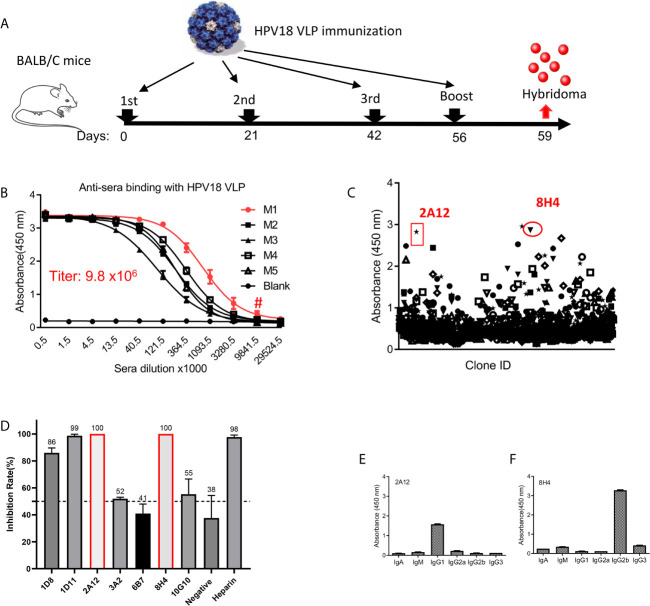
Generation of mAbs against HPV18. **(A)** The experimental schedule of immunization. **(B)** The titer of anti-sera was evaluated after the 3rd immunization in mice receiving HPV18 VLP. Y-axis represents the absorbance at 450 nm, and X-axis is the anti-sera dilution fold. Anti-sera from five immunized mice labeled with M1-5 were tested, and serum from non-immunized mice (Blank) were taken as a negative control. M1 presents the best binding (red line), a titer of 9.8 × 10^6^ dilution as indicated by #. **(C)** The summary of hybridoma supernatant binding with HPV18 VLP astested by ELISA. Each dot represents the binding of the supernatant from one culture well containing at least one hybridoma. Two dots, shown by red circle, indicate the parental clones of 2A12 and 8H14, respectively. **(D)** Top seven binders in **(C)** inhibiting HPV infection, each dot represents one hybridoma supernatant. Cell supernatant and heparin were taken as negative and positive controls, respectively. Two clones present complete inhibition were highlighted with red frame line. Subtype of 2A12 **(E)** and 8H4 **(F)** was tested by Subtype identification kit. Data of **(B, D–F)** represent mean ± SEM. All experiments of **(B–F)** were repeated twice.

Balb/c mice were immunized with HPV18 VLP generated in 293TT cells as depicted in **Figure 1A**. High titer (ranging 1.0–9.8 × 10^6^ dilution) anti-sera from five immunized mice specific for HPV18 VLP protein was achieved after the third immunization (**Figure 1B**). M1 mouse with the highest anti-serum titer was sacrificed for the hybridoma production. 1223 hybridoma supernatants, including 3,810 clones, were evaluated for the binding with HPV18 VLP protein, and, among them, 106 positive supernatants were scored as high positive (OD_450_ nm >1.0) for HPV18 VLP binding, yielding an overall hit rate of 8.7% (**Figure 1C**). Specially, hybridomas with high antigen binding were advanced for further subclone. Supernatants of seven mAbs were screened for preliminary neutralization assessment against HPV18 infection. Two monoclonal antibodies (named as 2A12 and 8H4) showed 100% inhibition against HPV18 infection (**Figure 1D**), and were selected for further characterization. 2A12 and 8H4 belong to IgG1 and IgG2b subtypes, respectively, as characterized by a subtyping kit (**Figures 1E, F**). Overall, Two mAbs with complete inhibition of HPV18 infection were isolated.

### Binding Characterization of Downselected Antibodies

**Figure 2:**
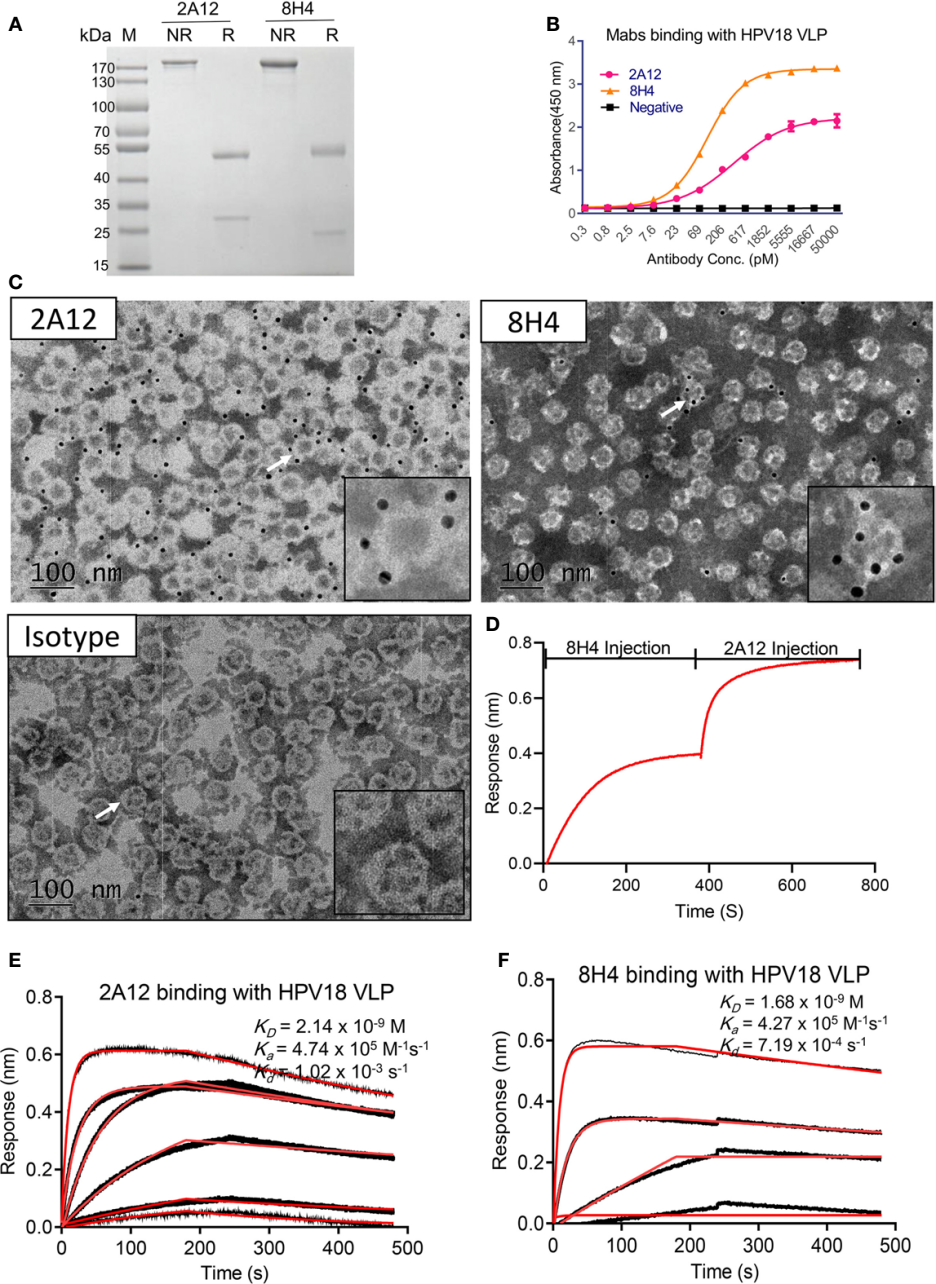
Characterization of 2A12 and 8H14. **(A)** The purity of 2A12 and 8H4 was determined by SDS-PAGE under non-reducing (NR) or reducing condition (R). **(B)** Serially diluted 2A12 and 8H4 monoclonal antibody binding with HPV18 VLP was analyzed by ELISA. Data represent mean ± SEM. **(C)** Immune electronic microscopy negative staining image showing HPV 18 VLPs recognized by 2A12, 8H4 and isotype control as indicated. The insets are enlarged images of individual VLP as indicated by corresponding arrows. Black dots are antibody conjugated with 10 nm colloidal gold particles. Gray circles with the white ring are the VLPs. The bar indicates 100 nm. **(D)** Epitope specificity analysis of 8H4 and 2A12 by BLI. HPV18 VLP was coated on the sensor, 8H4 antibody was added to bind for 400 s, followed by the addition of 2A12 for another 400 s. Kinetic binding curve of 2A12 **(E)** and 8H4 **(F)** with HPV18 VLP. Binding curves are colored black, and fit of the data to a 1:1 binding model is colored red. All experiments were repeated twice.

To further characterize the selected monoclonal antibodies, purified 2A12 and 8H4 were prepared and verified by SDS-PAGE (**Figure 2A**). ELISA results showed that 2A12 and 8H4 exhibited high binding with HPV18 VLP with EC_50_ values of 357 and 100 pM, respectively (**Figure 2B**). The binding of 2A12 and 8H4 to whole VLP was investigated by immune-electronic microscopy (Immune-EM). Dark dots of 10 nm colloidal gold particles conjugated with 2A12 or 8H4 but not the murine antibody (isotype control) surrounded the outer region of VLP, indicating that 2A12 and 8H4 recognized the HPV18 VLP (**Figure 2C**). 2A12 and 8H4 showed no reactivity with reduced HPV18 VLP as detected by Western-Blot (**Supplemental Figure 1**), suggesting that 2A12 and 8H4 epitopes are highly structure dependent. 2A12 and 8H4 were next evaluated for epitope specificity by bio-layer interferometry (BLI) using HPV18 VLP proteins as capture antigens. The antigens captured on AR2G biosensors were bound saturate concentrations (20 μg/ml) of 8H4 that were followed by 2A12 as the competing antibody at a concentration of 20 μg/ml. Only antibodies that bind to a non-competing site would be detected in the assay. The results revealed that 2A12 could still bind HPV 18 VLP even when HPV18 VLP was saturated by 8H4, suggesting that 2A12 and 8H4 react with distinct epitopes (**Figure 2D**). Purified 2A12 and 8H4 were further analyzed for HPV18 VLP binding kinetics by BLI. 2A12 and 8H4 bound HPV18 VLP with K_D_ values of 2.14 and 1.68 nM, respectively (**Figures 2E, F**). In summary, 2A12 and 8H4 recognize quaternary and non-overlapping epitopes with high affinity.

### Binding Specificity of 2A12 and 8H4 With HPV VLPs

**Figure 3:**
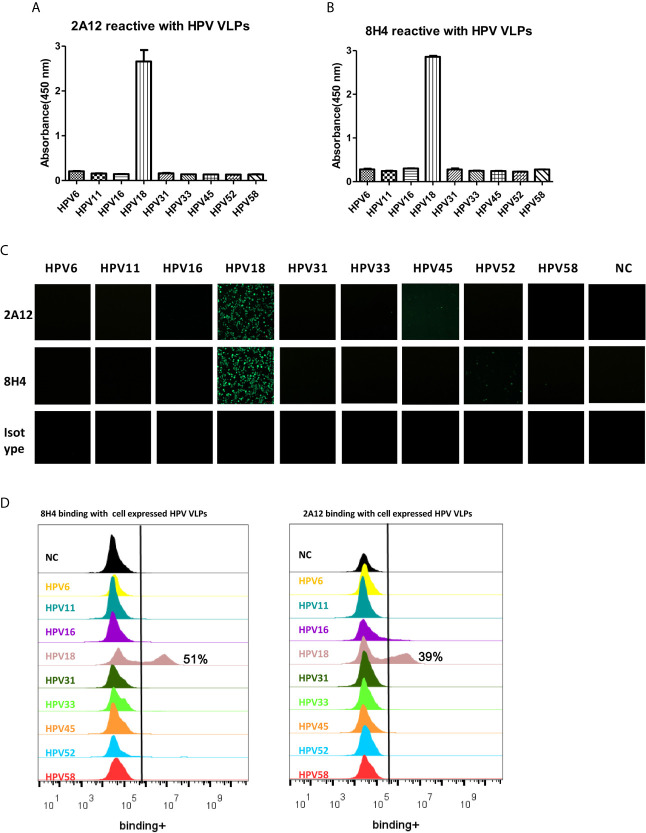
Characterization of binding specificity of 2A12 and 8H4. Analysis of 2A12 **(A)** and 8H4 **(B)** binding with VLPs derived from various subtypes of HPV by ELISA. Data represent mean ± SEM. **(C)** 2A12 and 8H4 binding with various subtypes of HPV VLPs detected by immunofluorescence assay. Isotype control antibody (Isotype) was taken as a negative control. **(D)** 2A12 and 8H4 binding with various subtypes HPV VLPs detected by FACS. All experiments were repeated twice.

The cross-reactivity of 2A12 and 8H4 against VLPs from nine common types of HPV, including HPV6, 11, 16, 18, 31, 33, 45, 52, and 58 was examined by ELISA, cell immunofluorescence and flow cytometry. ELISA showed that 2A12 and 8H4 reacted only with HPV18 VLP (**Figure 3A**). The specific binding was further validated by cell immunofluorescence and flow cytometry (**Figures 3B, C**). Altogether, these results indicate that 2A12 and 8H4 recognize respective epitopes that only present on HPV18 VLP.

### 2A12 and 8H4 Exhibited Excellent Neutralizing Potency Against HPV18

**Figure 4:**
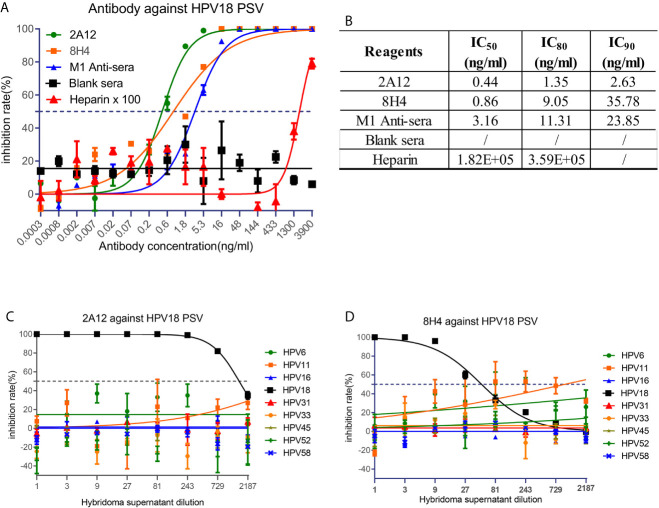
Neutralization activity of 2A12 and 8H4. **(A)** Neutralization activity of mAbs and mouse sera against HPV18 pseudovirus infection. Heparin was taken as a positive control and heparin ×100 represents that the concentration of heparin in use is the indicated concentration at *X* axis multiply 100 fold. **(B)** Summary of the neutralization titers (IC_50_, IC_80_ and IC_90_) against HPV18 pseudovirus infection. Neutralization activity of 2A12 **(C)** and 8H4 **(D)** against the pseudovirus infection of various HPV subtypes. Data represent mean ± SEM. All experiments were repeated twice.

To examine the neutralizing activity of 2A12 and 8H4, HPV pseudovirus neutralization experiments were performed. 2A12 and 8H4 were able to completely neutralize HPV18 infection with high potency at IC_50_ of 0.44 and 0.86 ng/ml, respectively (**Figures 4A, B**). Expectedly, 2A12 and 8H4 failed to neutralize the infection of any other eight common HPV subtypes (**Figures 4C, D**), consistent with their binding specificities (**Figure 3**). Heparin (H4784, Sigma-Aldrich), a drug used in the treatment of HPV infection (31), was used as the positive control with an IC_50_ of heparin at 1.82 × 10^5^ ng/ml. In other words, compared to the heparin control, 2A12 and 8H4 exhibited at least five orders of magnitude more potent neutralizing activity against HPV18 infection.

### Functional Activity of Humanized 2A12

**Figure 5:**
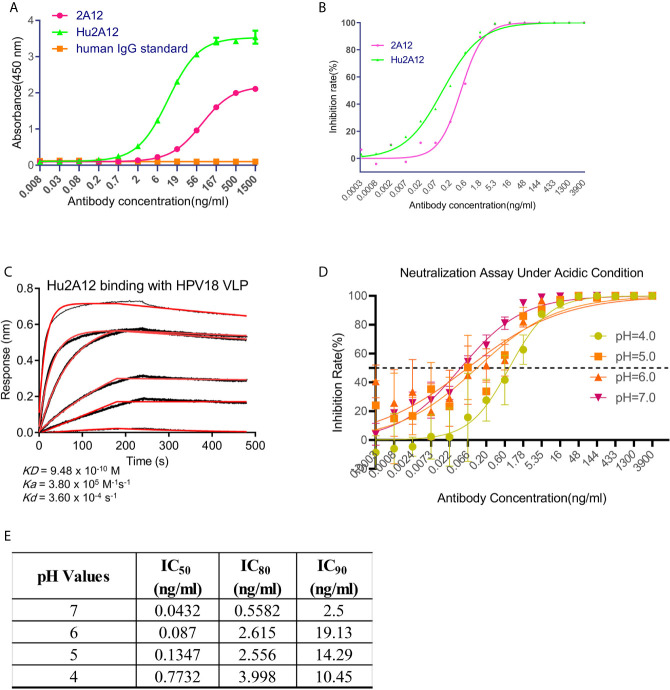
Characterization of humanized 2A12. **(A)** ELISA analysis of the reactivity of 2A12 and humanized 2A12 (Hu2A12) with HPV18 VLP. PBS binding with sGn served as a control (Blank). Data represent mean ± SEM **(B)** Neutralization activity of Hu2A12 against HPV18 pseudovirus infection. Data represent mean ± SEM. **(C)** Kinetic binding curve of Hu2A12 with HPV18 VLP. Binding curves are colored black, and fit of the data to a 1:1 binding model is colored red. **(D)** Neutralization activity of Hu2A12 against HPV18 pseudovirus infection under various pH. Different color curves represent different pH. **(E)** The summary of the neutralization titers (IC_50_, IC_80_ and IC_90_) against HPV18 pseudovirus infection in **(D)**. Data represent mean ± SEM. All experiments were repeated twice.

Given that 2A12 exhibited more potent neutralization against HPV18 infection with IC_90_ of 2.63 ng/ml compared to that conferred by 8H4 with IC_90_ of 35.78 ng/ml (**Figures 4A, B**). As a murine antibody, 2A12 will pose potential risk of immunogenicity when applied in human use and, therefore, needs to be humanized for clinical development. Surprisingly, humanized 2A12 (Hu2A12) exhibited improved binding with HPV18 VLP with an EC_50_ of 74.92 vs 492 pM for the parental 2A12 (**Figure 5A**). Consistently, Hu2A12 exhibited improved neutralization activity against HPV18 with an IC_50_ of 0.11ng/ml as compared to 2A12 with an IC_50_ of 0.44ng/ml (**Figure 5B**). The improved binding and neutralization activity were substantiated by the increased affinity of Hu2A12 to HPV18 VLP, with the *K_D_* of 0.95 nM vs. 2.1 nM for 2A12 (**Figures 2D**, **5C**). Together, these results indicate that Hu2A12 has higher affinity and improved neutralization potency than the parental antibody (**Figures 5B**).

Given the fact that vaginal environment is acidic and HPV neutralizing antibodies topically applied must sustain the degradation, the potency of Hu2A12 against HPV18 in various acidic pH settings was evaluated. IC_50_ values in pH 4.0, 5.0, 6.0 and 7.0 were 0.77, 0.134, 0.08 and 0.04 ng/ml, respectively (**Figures 5D, E**), showing the decreasing trend as the pH dropped though still highly potent with an IC_50_ of 0.77 ng/ml at pH4.0 (**Figures 5D, E**). Given the pH values ranged from pH 4.0 to 6.0 in the vaginal cervix (32), such acidic environment may have a limited impact on the neutralizing activity of Hu2A12. Together, these data indicates that Hu2A12 could retain neutralizing activity as a topical agent for the treatment of HPV cervical infection.

### *In Vitro* and *In Vivo* Characterization of Hu2A12-Hydrogel Formulation

**Figure 6:**
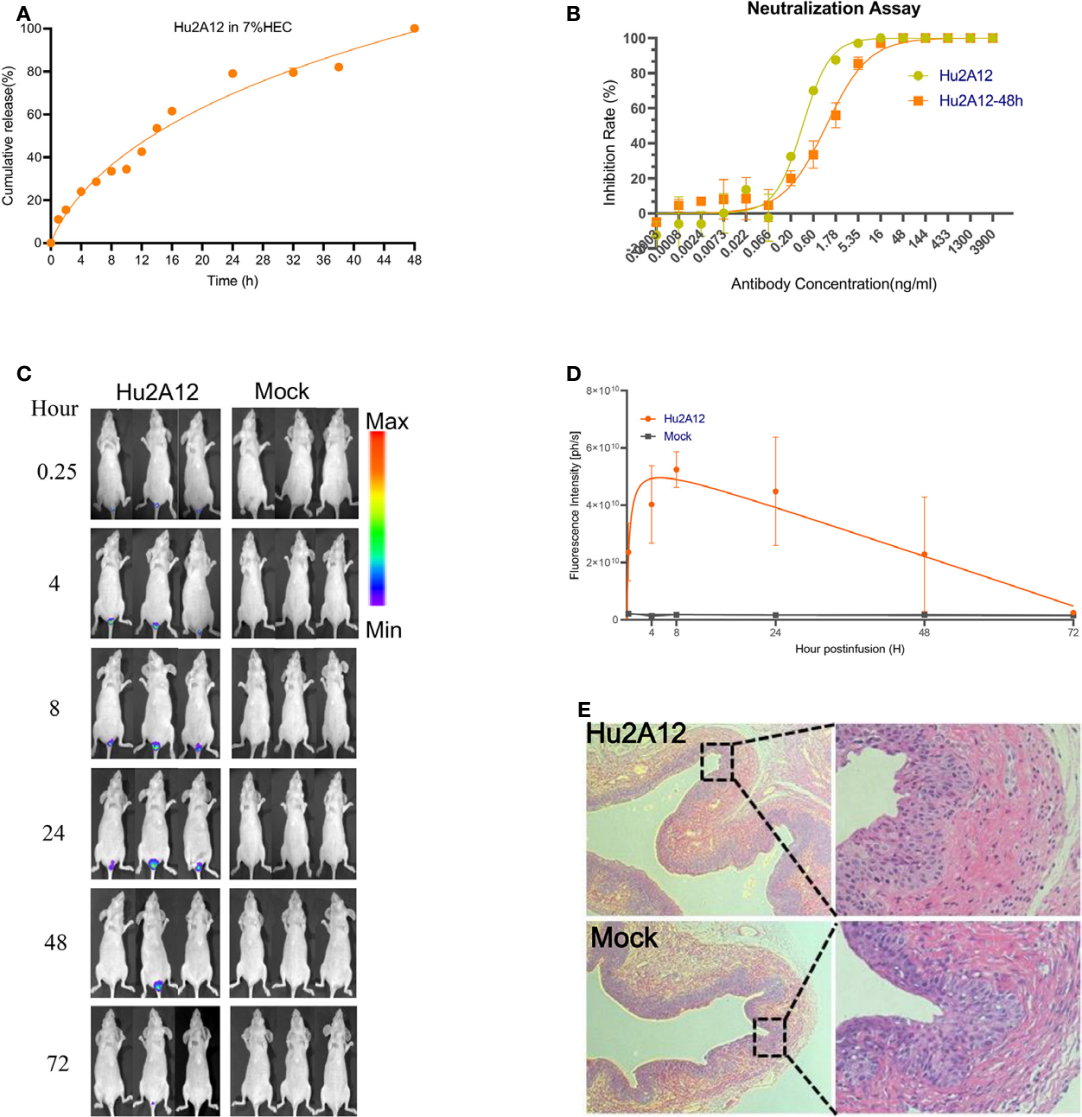
Characterization of Hu2A12 in gel. **(A)** The release of Hu2A12 in gel was determined at the indicated time. Data represent mean ± SEM. **(B)** Neutralization activity of Hu2A12 and Hu2A12 released from the gel at 48 h (Hu2A12-48h). Data represent mean ± SEM. **(C)** Sequential *in vivo* imaging of Hu2A12-750 conjugated with dye YF750 injected into the vagina of nude mice. **(D)**. Fluorescence intensity (ph/s) of mice shown in **(C)**. Data represent mean ± SEM. **(E)** Representative vaginal sections of mice from Hu2A12 in hydrogel and Mock group in **(B)** were analyzed by H&E staining. The right panel is enlarged images as indicated by rectangle.Images were visualized under ×10 objective lens. All experiments of **(B, D, E)** were repeated twice.

Hu2A12 was formulated in hydrogel as a topical agent to increase vaginal retention and improve release. Hu2A12 in the hydrogel was completely released within 48 hours and the released Hu2A12 retained comparable neutralizing activity, indicating that Hu2A12-hydrogel could be applied to treat HPV18 infection as vaginal biopharmaceutical agent (**Figures 6A, B**). To measure the kinetics of Hu2A12 in the form of hydrogel, hydrogel containing 2A12 was applied to mouse vaginal and the release of Hu2A12 was monitored at various time points. Hu2A12 could be observed in mouse vaginal within 48 h (**Figures 6C, D**), consistent with the released results of *in vitro* experiment as shown in **Figure 6A**. H&E-stained vaginal tissues of the above mice showed that Hu2A12 in hydrogel did not induce infiltration of inflammatory cells in the vaginal sections compared to the Mock control (**Figure 6E**), suggesting that Hu2A12 in hydrogel induced no toxic to vaginal tissue as topical agents. Altogether, Hu2A12-hydrogel could be released and retained anti-viral activity against HPV infection in the vaginal cervix.

## Discussion

Current prophylactic HPV vaccines have achieved significant prevention against HPV infection. However, a large number of women still fail to receive prophylactic HPV vaccines due to various reasons, such as costs, availability, or nonresponding to vaccination. In 2018, there were 570,000 reported new cases and 311,000 related deaths, suggesting that effective medical treatment of HPV infection is urgently needed.

Persistent HPV infection is the main risk factor and a pre-requisite for the development of the premalignant conditions of cervical intraepithelial neoplasia or adenocarcinoma *in situ*. Therefore, neutralizing antibodies can be used to block the released virus from infecting nascent epithelial cells, thus inhibiting the malignant transformation of the cells. Neutralizing antibodies have been widely applied to treat viral infections caused by RSV, Ebola, HIV, SAR-CoV-2 etc. However, the development of HPV neutralizing antibodies is very rare. Thus our potent neutralizing antibody, Hu2A12, will be a potential candidate therapeutic agent for the topical treatment of HPV18 infection.

Considering the repeated cycles of HPV release and infection in the cervical basal epithelium, topical application of neutralizing antibodies will be a plausible approach to stop the HPV infection, thus preventing malignant transformation of the epithelium. Acid stable Hu2A12 will offer a valuable advantage for its application at the vaginal/cervical environment. In addition, the slow-release and the retention of bioactivity of Hu2A12 in hydrogel formulation offer additional benefits for using the antibody as a topical agent to treat HPV18 infection. Unlike vaccines, which usually take weeks to generate immune protection in vaccinated individuals, neutralizing mAbs as topical agents may provide immediate protection against viral infection, and are thus suitable for people at all ages and particularly suitable for high-risk populations and immunocompromised individuals who typically do not generate sufficient nAbs after vaccination. Furthermore, topical agents could release high concentration of potent neutralizing mAbs at the HPV infected sites compared to the systemic neutralizing mAbs elicited by HPV vaccine. In the developing world, even if the vaccines are available it would take many years to build up enough coverage of the populations under risk; therefore, in the foreseeable future women will continue to be infected and effective treatment drugs will be needed. Altogether, compared to the success in the prevention of HPV infection achieved by current licensed HPV vaccines, topical hydrogels containing anti-viral agents may provide immediate treatment for people without receiving HPV vaccines or who fail to mount antibody immunity after vaccination.

In this study, two potent neutralizing antibodies, 2A12 and 8H4 were isolated from hybridomas prepared from the mice immunized with HPV18 VLP. Both 2A12 and 8H4 could completely neutralize HPV18 infection with high potency with IC_50_ values of 0.44 to 0.86 ng/ml (**Figures 4A, B**). To our knowledge, these two antibodies are the most potent mAbs against HPV18 reported. Furthermore, 2A12 and 8H4 recognize distinct quaternary epitopes and exhibit highly specific binding with HPV18. Hu2A12 exhibited improved neutralizing activity against HPV18 infection and its neutralization activity was not affected in various acidic pH settings and in hydrogel (**Figures 5D, E**), suggesting that Hu2A12 can be a promising candidate for topical therapeutic agent against HPV18 infection.

Several topical therapies for HPV or CIN, including immune-modulators, anti-proliferative medications, antivirals, hormones, and herbal/alternative therapies are in various stages of clinical trial (11). Nevertheless, studies of antibodies as topical agents against HPV have been limited due to the difficulty in isolating antibodies with potent neutralizing activities, the challenge in delivering antibodies to the infection sites in the basal layer of the cervical epithelium and the efficacy of antibodies eliminating infected cells. The development of Hu2A12 will open a new therapeutic avenue for neutralizing antibodies as topical agents in treating other high-risk HPV infections such as HPV16, 31, 33, 45, 52, 58 etc.

The main limitation of this study is not evaluating the *in vivo* efficacy of Hu2A12-hydrogel in the treatment of HPV18 infection due to the lack of a suitable small animal model. Clinical trials demonstrated that robust neutralizing antibodies against HPV in serum and in cervicovaginal secretions were correlated to protection (33, 34), suggesting that human studies in the evaluating Hu2A12 as topical agents will be merited.

In summary, Hu2A12 with high neutralization potency against HPV18 was developed. The efficacy, delivery, safety, and accessibility of Hu2A12 were characterized, indicating that Hu2A12 will be promising as a topical agent to treat HPV18 infection.

## Data Availability Statement

The raw data supporting the conclusions of this article will be made available by the authors, without undue reservation.

## Ethics Statement

The animal study was reviewed and approved by the Committee on the Use of Live Animals by the Ethics Committee of Nanjing Drum Tower Hospital.

## Author Contributions

All authors contributed to the work fulfilled the criteria adopted from ICMJE. BH, LZ, and XW conducted most experiments, analyzed the data, and wrote the draft manuscript. HW, HS, DZ, HY, NZ, SX, WN, and YH provided technical assistance and did animal experiments. XW and ZW designed the study, monitored and financially supported the study, and revised the manuscript. All authors contributed to the article and approved the submitted version.

## Funding

This work was supported by The Major Research and Development Project (2018ZX10301406 to ZW), National Science Foundation of China (NSFC) (No. 81803414 to XW, 31970149 to ZW), Nanjing University-Ninxia University Collaborative Project (Grant# 2017BN04 to ZW), Research Foundation of JiangSu Commission Health project (Grant# ZDA2020014 to XW), and Jiangsu province “Innovative and Entrepreneurial talent” and Six Talent Peaks Project of Jiangsu Province.

## Conflict of Interest

Author LZ was employed by the company Abrev Biotechnology Co., Ltd. Author HS was employed by Y-Clone Medical Science Co. Ltd. A patent application on 2A12 was submitted by Y-Clone Medical science Co. Ltd., under CN201911358261X.

The remaining authors declare that the research was conducted in the absence of any commercial or financial relationships that could be construed as a potential conflict of interest.
