# Supplementary material for: Potent Neutralizing Humanized Antibody With Topical Therapeutic Potential Against HPV18-Related Cervical Cancer
Source: Front Immunol. 2021 Jun 24;12:678318. doi: 10.3389/fimmu.2021.678318 (PMC8264373; doi:10.3389/fimmu.2021.678318)
Supplement: Supplementary file 1 [file DataSheet_1.docx]

**Supplemental Materials for**

**Potent neutralizing humanized antibody with topical therapeutic potential against HPV18-related cervical cancer**

Bilian Huang ^1^†, Linjing Zhu ^2^†, Hongxia Wei^3^, Haixia Shi^4^, Doudou Zhang^2^, Huanyun Yuan^2^, Linlin Luan^2^, Nan Zheng^1^, Shijie Xu^2^, Waqas Nawaz^1^, Ying Hong^5^*, Xilin Wu^1,2^*, Zhiwei Wu ^1, 6, 7, 8*^

*1.Center for Public Health Research, Medical School, Nanjing University, Nanjing, P.R. China.*

*2. Department of antibody, Abrev Biotechnology Co., Ltd. Nanjing, P.R. China.*

*3. Department of Infectious Disease, The second Hospital of Nanjing, Nanjing University of Chinese Medicine, Nanjing, P.R. China.*

*4. Department of Antibody, Y-clone Medical science Co. Ltd. Suzhou, P.R. China.*

*5. Obstetrics and Gynecology Department, Nanjing Drum Tower Hospital, Affiliated Hospital of Nanjing University Medical School, Nanjing, China.*

*6. School of Life Sciences, Ningxia University, Yinchuan, P.R. China.*

*7. Jiangsu Key Laboratory of Molecular Medicine, Medical School, Nanjing University, Nanjing, P.R. China.*

*8. State Key Laboratory of Analytical Chemistry for Life Science, Nanjing University, Nanjing, P.R. China.*

*Corresponding author: Z. Wu, E-mail: [wzhw@nju.edu.cn](mailto:wzhw@nju.edu.cn), X. Wu, E-mail: [xilinwu@nju.edu.cn](mailto:xilinwu@nju.edu.cn) Y. Hong, E-mail: hongying@nju.edu.cn.

Mailing address: School of Life Sciences, Ningxia University, Yinchuan, 750021, China. Phone: +86 (25) 8368-6092. Fax: +86 (25) 8359-6023.

†These authors contributed equally to this work.

**Supplemental figures**

**
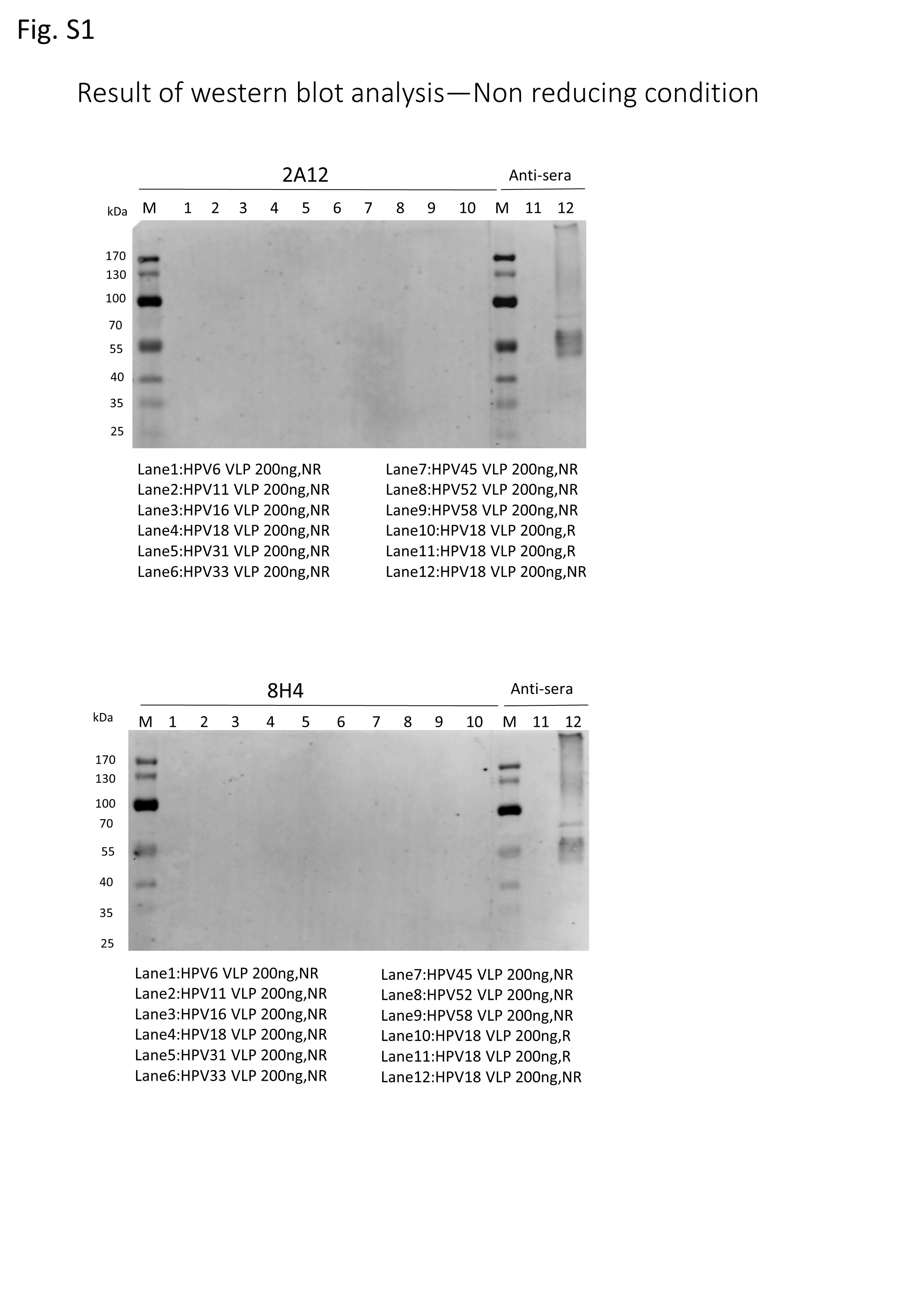
**

**Supplemental Figure 1. VLPs binding to 2A12 and 8H4.** VLPs from various HPV types under reducing conditions (R) or nonreducing conditions (NR) was detected by western-blot with 2A12 (A) and 8H4 (B). Mouse polyclonal anti-sera specific (Anti-sera 1:10000 dilution) for HPV 18 VLP was taken as positive control.
